# Digitalisierung in Angeboten der Erwachsenenbildung/Weiterbildung

**DOI:** 10.1007/s40955-021-00185-4

**Published:** 2021-09-16

**Authors:** Regina Egetenmeyer, Stefanie Kröner, Anne Thees

**Affiliations:** 1 Julius-Maximilians-Universität Würzburg, Würzburg, Deutschland; 2 Hochschule Reutlingen, Reutlingen, Deutschland

**Keywords:** Angebote, Gelingensbedingungen, Digitalisierung, Erwachsenenbildung, Weiterbildung, Offers, Conditions for success, Digitalisation, Adult education, Continuing education

## Abstract

Digitalisierung und Mediatisierung prägen die Gesellschaft und auch die Erwachsenenbildung/Weiterbildung. Der Beitrag geht der Frage nach, wie Digitalisierung in Angeboten der Erwachsenenbildung/Weiterbildung gelingt. Damit wird ein Fokus auf den Einsatz digitaler Medien gelegt. Dazu werden die Angebotsentwicklung für Adressatinnen und Adressaten sowie Teilnehmende, medienbezogene Inhalte, Lehr- und Lernarrangements mit digitalen Medien, der Einsatz digitaler Medien und die Zugänglichkeit von Lehr- und Lernmaterialien als relevante Merkmale identifiziert. Insgesamt zeigen die analysierten Interviewdaten, dass der Einsatz digitaler Medien in Angeboten eine Erweiterung der didaktischen Aufgaben darstellt, da Angebote mit digitalen Medien zielgenau auf die Bedarfe und Möglichkeiten von Adressatinnen und Adressaten sowie Teilnehmenden abgestimmt werden müssen.

## Abstract (en)

Digitalisation and mediatisation shape society and adult and continuing education as well. This article follows the question how digitalisation in offers of adult and continuing education succeeds. For this purpose, the development of offers for recipients and participants, media content, teaching and learning settings with digital media, use of digital media and the accessibility of teaching and learning material have been identified as relevant characteristics. Overall, the analysed interview data show that digitalisation in offers are an extension to didactical offers as offers with digital media accurately have to be compatible with the needs and possibilities of recipients and participants.

## Einleitung

Digitalisierung und Mediatisierung prägen die Gesellschaft in einem erheblichen Ausmaß. „Digitalisierung“ wird als ein Wandlungsprozess verstanden, der durch die Verwendung digitaler Informations- und Kommunikationstechnologien bestimmt wird (Tulodziecki et al. 2019). Damit erfährt der ursprüngliche Begriff „Digitalisierung“ als Umwandlung analoger Größen in direkte bzw. binäre Werte oder digitale Repräsentationen (Knaus 2016, S. 101f.) eine Erweiterung. In Anlehnung an Krotz (2016) wird im vorliegenden Beitrag davon ausgegangen, dass auf digitaler Technik beruhende Mediatisierungsprozesse zu einem Wandel sozialer Situationen führen. In sogenannten „mediatized worlds“ (Hepp und Krotz 2014, S. 11) sind Kommunikation und Medien eng miteinander verzahnt. Medien verändern sich stetig und führen zu kontinuierlichen kulturellen und sozialen Veränderungen. „Mediatisierung“ wird als ein Konstrukt verstanden, das aus verschiedenen Phänomenen besteht. Über die anhaltende Veränderung von Kommunikation und Technik hinaus sind die Konsequenzen der Aneignung der unterschiedlichen Medien auf den Menschen und seine Umwelt zu betrachten (Krotz 2007, S. 12).

In diesem Sinne prägen Digitalisierung und Mediatisierung auch Angebote der allgemeinen Erwachsenenbildung und beruflichen Weiterbildung^1^. Als Teil von Programmen der Erwachsenenbildung/Weiterbildung können in Angeboten veränderte Bedarfe von Teilnehmenden beobachtet werden, die sich in der inhaltlichen und didaktischen Angebotsentwicklung zeigen. Dadurch entstehen Anforderungen an Mitarbeitende, Mitwirkende und Dozierende, diesen Bedarfen gerecht zu werden.

Digitalisierung und Mediatisierung haben jedoch nicht nur Einfluss auf die Angebotsentwicklung, sondern auch auf Einrichtungen der Erwachsenenbildung/Weiterbildung als Organisationen. Auch hier entstehen neue Anforderungen, wie beispielsweise die Digitalisierung von Verwaltungsprozessen oder Veränderungen in der Arbeits- und Organisationskultur. Im vorliegenden Beitrag wird deshalb bei der Verwendung des Begriffs „Digitalisierung“ immer die Perspektive der Mediatisierung mitgedacht. Dies bedeutet beispielsweise, dass Digitalisierung in der Erwachsenenbildung/Weiterbildung nicht nur die Einführung und Anwendung digitaler Medien und Technik bedeutet. Vielmehr hat die Anwendung und Nutzung von digitalen Medien und Technik auch Folgen mit Blick auf die Art und Weise der Zusammenarbeit und die Gestaltung von Lehr- und Lernsettings. Sie hat beispielsweise Einfluss auf die Art des Angebots, die Ansprache und Identifikation von Adressatinnen und Adressaten. Digitalisierungsentwicklungen in Angeboten sind als eingebettet in verschiedene Ebenen der Erwachsenenbildung/Weiterbildung (Gesellschaft; Dachorganisationen; Organisationen; Angebote und Programme; Mitarbeitende, Dozierende und Mitwirkende; Teilnehmende) zu verstehen (z. B. Tietgens 1992; Schmidt-Lauff 2012; Grotlüschen 2018; Egetenmeyer et al. 2019).

Digitalisierung und Mediatisierung stellen die Entwicklung von Angeboten in der Erwachsenenbildung/Weiterbildung deshalb vor neue Herausforderungen. Vor diesem Hintergrund geht der Beitrag der Frage nach, wie Digitalisierung in Angeboten der Erwachsenenbildung/Weiterbildung gelingt. Dazu erfolgt zunächst eine Einführung in den Diskurs um Angebote in der Erwachsenenbildung/Weiterbildung. Dieser wird eingebettet in den Diskurs um Programme in der Erwachsenenbildung/Weiterbildung. Zudem werden unter Rückgriff auf die Medienpädagogik Perspektiven der Digitalisierung von Angeboten identifiziert. Für die Untersuchung der Fragestellung wird auf Daten aus Gruppendiskussionen und Einzelinterviews zurückgegriffen, die im Projekt Digitalisierung in der Erwachsenenbildung und beruflichen Weiterbildung (DigiEB 2019 bis 2021) erhoben wurden. Für die Analyse der Daten werden die Merkmale Angebotsentwicklung für Adressatinnen und Adressaten sowie Teilnehmende, medienbezogene Inhalte, Lehr- und Lernarrangements mit digitalen Medien, Einsatz digitaler Medien und Zugänglichkeit von Lehr- und Lernmaterialien untersucht. Abschließend werden zu diesen Merkmalen Gelingensbedingungen identifiziert. Diese basieren auf der Gelingenseinschätzung der Interviewpersonen mit Blick auf die Gestaltung von Angeboten der Erwachsenenbildung/Weiterbildung. Der Beitrag gibt damit einen Einblick in die Gestaltungsperspektiven der Interviewpersonen.

## Angebote und Merkmale der Digitalisierung

### Angebote in der Erwachsenenbildung/Weiterbildung

Der Begriff „Angebot“ ist in den erwachsenenpädagogischen Diskurs um den Begriff „Programme“ einzubetten. „Programme“ werden in Anlehnung an Gieseke (2015) als „zeitgeschichtlich materialisierte(n) Ausdruck gesellschaftlicher Auslegung von Erwachsenenbildung durch einen bestimmten Träger, realisiert über eine Vielzahl von Angeboten“ (S. 165) verstanden. Im Unterschied zu anderen Bildungsbereichen sind Angebote in der Erwachsenenbildung/Weiterbildung überwiegend nicht an curriculare Regelungen gebunden. Vielmehr antworten Programme und Angebote dynamisch auf jeweilige gesellschaftliche Entwicklungen, bildungspolitische Rahmenbedingungen, ökonomische Erwartungen sowie auf Nachfragen und Bedarfe von Teilnehmenden und Unternehmen. Gieseke und Enoch (2011, S. 7) verweisen auf die „rhizomartige Entwicklung“ von Programmen, deren Entwicklung ein offener Prozess ist. Es bilden sich vorläufige Strukturen, die sich laufend verändern. Ein Programm besteht aus mehreren Angeboten (Gieseke 2018). Die einzelnen Angebote können verschiedenen Programmbereichen (z. B. Sprache, kaufmännische Aufstiegsfortbildungen, Religion und Gesellschaft) zugeordnet werden (ebd., S. 19–20). Angebote und Programme sind in der Erwachsenenbildung/Weiterbildung in Ziele und Leitbilder der Einrichtungen sowie deren Träger eingebettet. Während sich der Begriff „Programm“ häufig im Kontext von Volkshochschulen findet, wird der Begriff „Angebot“ auch dort genutzt, wo eher Einzelangebote ohne Programmstrukturen bereitgestellt werden. Im vorliegenden Beitrag wird der Begriff „Angebot“ genutzt, um Digitalisierungsentwicklungen in Einzelangeboten zu untersuchen. Jedoch werden Angebote als eingebettet in Programme von Einrichtungen der Erwachsenenbildung/Weiterbildung verstanden.

Programme in der Erwachsenenbildung/Weiterbildung sind „Ergebnis pädagogisch-professionellen Handelns“ (Von Hippel 2017, S. 199) von Programmplanenden. Für dieses Handeln liegen in der Erwachsenenbildungsforschung verschiedene Modelle vor. Dazu zählen z. B. Programmplanung als Angleichungshandeln (Gieseke 2003, 2008), Gestaltung professioneller Antinomien (Von Hippel 2011) oder das Mehrebenenmodell (Schrader 2011; von Hippel 2017).

Ob und in welcher Weise gesellschaftliche Entwicklungen der Digitalisierung in Angeboten zum Ausdruck gebracht werden, ist abhängig von Entscheidungen und professionellen Handlungen in der Programmplanung. Damit werden Fragen zur Digitalisierung in Angeboten im vorliegenden Beitrag als eine Frage von erwachsenenpädagogischer Professionalisierung verstanden (Breitschwerdt und Egetenmeyer im Druck).

### Merkmale der Digitalisierung in Angeboten in der Erwachsenenbildung/Weiterbildung

Mit Blick auf die Angebote von Erwachsenenbildung/Weiterbildung bezieht sich der Begriff „Digitalisierung“ vor allem auf digitale Medien. Hierbei geht es jedoch nicht nur um den Einsatz digitaler Medien, sondern auch um die Fragen, wie Angebote angemessen für Adressatinnen und Adressaten entwickelt werden können, worauf sich Inhalte und Ziele von Angeboten richten, wie Lehr- und Lernarrangements didaktisch gestaltet werden können und wie der Zugang zu digitalen Lehr- und Lernmaterialien gewährt wird. Digitale Medien werden im vorliegenden Beitrag als eingeordnet in gesamtgesellschaftliche Digitalisierungs- und Mediatisierungsentwicklungen verstanden. Die Verwendung des Begriffs „Digitalisierung“ soll diese Einbettung anzeigen. Zudem soll sie auf die Einbettung von Angeboten in Programme, Einrichtungen und gesellschaftliche Entwicklungen verweisen. Mit Blick auf die Angebote können folgende Untersuchungsmerkmale bestimmt werden:

Das Merkmal **Angebotsentwicklung für Adressatinnen und Adressaten sowie Teilnehmende** richtet sich darauf, welche Adressatinnen und Adressaten sowie Teilnehmende mit welchen digitalen Medien erreicht werden können. Aus der Perspektive der Praxis stellt sich die Frage, welche digitalen Medien passgenau bei welchen Adressatinnen und Adressaten sowie Teilnehmenden eingesetzt werden können. Von Hippel (2007, S. 48ff.) verweist auf Mediennutzungsmotive, über die Teilnehmende gewonnen werden. Dazu zählt z. B. der Wunsch nach Unterhaltung, Austausch oder Information, der Teilnehmende dazu motiviert, an Angeboten mit digitalen Medien teilzunehmen. Für Einrichtungen der Erwachsenenbildung/Weiterbildung stellt sich die Herausforderung, digitale Medien passgenau für die verschiedenen Teilnehmenden einzusetzen: Welche digitalen Medien unterstützen die Motivation zur Teilnahme an einer Weiterbildung und welche stellen ein Hindernis für welche Adressatinnen und Adressaten dar? Bei der Auswahl technischer Werkzeuge sind die *Usability* (z. B. einfache Registrierung) (Reindl 2009; Engelhardt 2019, S. 170–171) und privaten Nutzungsoptionen von Adressatinnen und Adressaten mitzudenken. Cocquyt et al. (2017) verweisen darauf, dass vulnerable Gruppen, insbesondere Teilnehmende mit einer anderen Muttersprache, besonders von Online-Angeboten profitieren.

#### Medienbezogene Inhalte

Digitale Medien sind in verschiedener Weise seit langer Zeit Inhalte von Angeboten der Erwachsenenbildung/Weiterbildung (vgl. z. B. Von Hippel 2007). Zu differenzieren sind Medientechnik, Mediendidaktik und Medienbildung. „Medientechnik“ bezieht sich auf die Vermittlung von Anwendungswissen zu digitaler Technik, wie z. B. Office-Anwendungen. Der Begriff „Medienbildung“ eröffnet eine reflexive Perspektive auf Medien. Tulodziecki et al. (2019, S. 41; Tulodziecki 2011) unterscheiden zwischen dem Begriff „Medienbildung“ als „Lehren und Lernen über Medien“ und „Mediendidaktik“ als „Lehren und Lernen mit Medien“. Der Begriff „Medienbildung“ ist im schulpädagogischen Kontext eng mit dem Begriff „Medienerziehung“ verbunden. Mit Blick auf das Ziel der Entwicklung von kritischer Urteilsfähigkeit über Medien und einem emanzipatorischen Anspruch nutzen die Autorinnen und Autoren den Begriff „Medienbildung“ auch für die Schulpädagogik. „Medienbildung“ hat den Anspruch, Entwicklungen durch Digitalisierung kritisch zu begleiten sowie den persönlichen kritischen Umgang mit digitalen Medien zu stärken. „Mediendidaktik“ richtet sich hingegen auf den selbstbestimmten, kritischen und kompetenten Einsatz von digitalen Medien durch Lehrpersonen in Lehr- und Lernsettings (Petko 2020, S. 23). Mit Blick auf die Inhaltsdimension der Digitalisierung von Angeboten in der Erwachsenenbildung wird deshalb unterschieden zwischen „Medientechnik“ als Inhalte zur Vermittlung von Fähigkeiten zur Medienverwendung; „Mediendidaktik“ als Inhaltsvermittlung mit Medien; und „Medienbildung“ als kritisch-emanzipatorische Inhaltsvermittlung über Medien.

#### Lehr- und Lernarrangements mit digitalen Medien

Mit Blick auf Lehr- und Lernarrangements kann unterschieden werden zwischen Präsenzangeboten, in denen digitale Medien eingesetzt werden, Online-Kursen, die ausschließlich virtuell stattfinden, und Blended-Learning-Angeboten als Kombination von Präsenz- und Online-Phasen. Der Einsatz digitaler Medien kann in allen Settings auf unterschiedliche Weise erfolgen. In Präsenzangeboten können verschiedene Medienarten (z. B. interaktive Boards), digitale Werkzeuge (z. B. Präsentationssoftware) oder inhaltlich-didaktisch strukturierte Medienangebote (z. B. Erklärvideos) eingesetzt werden (Tulodziecki et al. 2019). Zudem können analoge und digitale Medien (z. B. Flipcharts und digitale Projektion) kombiniert werden (Ladel 2018). Zu den Online-Kursen zählen beispielsweise Massive Open Online Courses (MOOCs) (Helbig und Hofhues 2018a, b; Sgier et al. 2018; Rohs und Ganz 2015; Walkling 2019). Das Angebot an rein virtuellen Seminaren, wie sie durch die Kontaktbeschränkungen im Rahmen der Covid-19-Pandemie erfolgen, stellt nur eine Form von digitalen Lehr- und Lernarrangements dar. Beispiel für ein Blended-Learning-Arrangement sind Flipped Classrooms, in denen die Vorbereitung auf die Sitzungen anhand (digitaler) Lernmaterialien erfolgt und diese im Präsenzgeschehen oder in virtueller Begegnung vertieft werden (Grote et al. 2015; Freisleben-Teutscher 2015; Buschle und König 2018; Baetge 2019; Distefano und Stein 2019).

#### Einsatz digitaler Medien

Digitale Medien in pädagogischen Settings können differenziert werden in digitale Medienarten, digitale Werkzeuge und didaktisch-strukturierte digitale Medienangebote (Tulodziecki et al. 2014; Breitschwerdt et al. angenommen). Digitale Medienarten beziehen sich auf die Hardware, wie z. B. Beamer mit Notebook oder interaktive Tafel. Sie sind als „Mittler zu verstehen, durch die in kommunikativen Zusammenhängen potenzielle Zeichen mit technischer Unterstützung aufgenommen bzw. erzeugt, verarbeitet, übertragen, gespeichert oder wiedergegeben bzw. präsentiert werden und verfügbar sind“ (ebd., S. 33). Digitale Werkzeuge hingegen beziehen sich auf Software, die ohne Inhalt und didaktische Struktur vorliegt, und deren didaktisch-inhaltliche Struktur entwickelt werden muss. Dazu zählen zum Beispiel Lernmanagementsysteme, Präsentationsprogramme oder Tools zur Erstellung virtueller Pinnwände. Davon wiederum sind didaktisch-strukturierte digitale Medienangebote zu unterscheiden, die „inhaltlich ausgerichtet und didaktisch strukturiert“ (Tulodziecki et al. 2019, S. 90) sind. Die Begriffskombination didaktisch-strukturierte digitale Medienangebote wird vom übergeordneten Begriff Angebote abgegrenzt. Zu den didaktisch-strukturierten digitalen Medienangeboten zählen beispielsweise eBooks, Lehrprogramme oder Lernspiele.

#### Zugänglichkeit von Lehr- und Lernmaterialien

Mit dem Einsatz von digitalen Medien hängt der Zugang von Teilnehmenden, Lehrenden und Einrichtungen zu digitalen Lehr- und Lernmaterialien zusammen. Open Educational Resources stellen didaktisch-strukturierte digitale Medienangebote dar, zu denen ein offener und kostenfreier Zugang ermöglicht wird. Sie können dazu beitragen, „Chancenungleichheiten ab[zu]bauen“ (Muuß-Merholz 2015, S. 14) und Teilhabe zu ermöglichen (Goertz 2019). Für Einrichtungen der Erwachsenenbildung/Weiterbildung stellt sich die Frage, wie die Entwicklung von Open Education Resources finanziert werden kann.

Der Blick auf die Angebote zeigt, dass sich Digitalisierung in der Angebotsentwicklung in Einrichtungen der Erwachsenenbildung/Weiterbildung auf die Auswahl, Nutzung, Entwicklung, den Zugang und die kritische Reflexion digitaler Medien bezieht. Diese Perspektiven stellen eine grundlegende Erweiterung der erwachsenenpädagogischen Angebotsplanung dar. Vor diesem Hintergrund geht der Beitrag der Frage nach, wie die Digitalisierung in Angeboten der Erwachsenenbildung/Weiterbildung gelingt.

## Zum methodischen Vorgehen

Die Untersuchung des Gelingens von Digitalisierung in Angeboten der Erwachsenenbildung/Weiterbildung basiert auf Daten eines Fallstudiendesigns (Ludwig 2005), die im Rahmen des BMBF-Projekts *Digitalisierung in der Erwachsenenbildung und beruflichen Weiterbildung* (2019–2021) erhoben wurden. Die Gesamtstudie orientiert sich an einer Mehrebenenperspektive, die folgende Ebenen im Blick hat: (1) Gesellschaft als Staat, Markt und Zivilgesellschaft, (2) Dachorganisationen, (3) Einrichtungen, (4) Programme und Angebote, (5) Personal, Mitwirkende und Dozierende und (6) Teilnehmende^2^. Der vorliegende Beitrag widmet sich ausschließlich der Ebene Programme und Angebote. Im Rahmen der Fallstudie werden sechs Einrichtungen und zwei dazugehörende Dachorganisationen aus der Erwachsenenbildung und beruflichen Weiterbildung untersucht. Dazu zählen zwei Einrichtungen, die außerbetriebliche berufliche Aufstiegs- und Anpassungsfortbildungen anbieten. Eine weitere Dachorganisation und zwei dazugehörige Einrichtungen sind im Bereich der kommunalen Erwachsenenbildung verankert, die allgemeine, politische und berufliche Erwachsenenbildung/Weiterbildung anbieten. Eine Dachorganisation und zwei dazugehörige Einrichtungen sind der konfessionellen Erwachsenenbildung zuzuordnen, die sowohl allgemeine, persönlichkeitsbildende, politische, als auch berufliche Angebote bereitstellen. Alle Falleinrichtungen wurden ausgewählt, da sie bereits über Erfahrungen in der digitalen Entwicklung von Angeboten in der Erwachsenenbildung/Weiterbildung verfügen und eine gewisse Affinität zum Thema Digitalisierung vorliegt. Die vorliegende Studie kann damit Aussagen zu einem inhaltlich relativ breiten Spektrum der klassischen Einrichtungen der Erwachsenenbildung/Weiterbildung treffen. Jedoch begrenzt sich die Aussagekraft der Analyseergebnisse auf Einrichtungen, die über eine hohe Affinität mit Blick auf den Einsatz digitaler Medien verfügen. Hinzu kommt, dass die Daten vor der Pandemie erhoben wurden und die Digitalisierungsentwicklungen seit Sommer 2020 nicht in den Interviewdaten berücksichtigt sind.

Methodologisch orientiert sich die Studie an der *Critical Communicative Method* nach Gómez et al. (2011) und einer gestaltungsorientierten Bildungsforschung nach Tulodziecki et al. (2014). Beiden Ansätzen gemeinsam ist eine dialogische Forschungsperspektive zwischen Forschenden sowie Akteurinnen und Akteuren des Untersuchungsfeldes. Grundannahme der Forschungsansätze ist, dass Forschung und Praxis unterschiedliche gewinnbringende Perspektiven auf den Untersuchungsgegenstand haben. Der Dialog erweitert die gegenseitigen Perspektiven auf den Untersuchungsgegenstand und trägt dazu bei, dass Forschungserkenntnisse so kommuniziert werden, dass sie einen Beitrag zur Praxisentwicklung leisten können. Eine Konsequenz dieser methodologischen Perspektive ist es, dass Forschungsfragen, Untersuchungsmerkmale und Ziele der Studie gemeinsam mit den Akteurinnen und Akteuren des Untersuchungsfeldes diskutiert und entwickelt werden, um so eine Vertiefung und Erweiterung des Forschungsgegenstandes zu erreichen. Dazu wurden Untersuchungsmerkmale anhand einer Literaturanalyse^3^ identifiziert. Diese wurden in acht Gruppendiskussionen von April bis August 2019 (je eine pro Falleinrichtung und Dachorganisation) vorgestellt und hinsichtlich ihrer Relevanz für die Praxis diskutiert. Dabei wurden die Interviewpersonen nach ihren Gelingenserfahrungen gefragt.

Für das vorliegende Papier werden folgende Untersuchungsmerkmale mit dazugehörigen Unter-Merkmalen identifiziert:

- *Angebotsentwicklung für Adressatinnen und Adressaten sowie Teilnehmende: *Erreichbarkeit, gruppenspezifische Erfahrungen mit digitalen Lernangeboten
- *Medienbezogene Inhalte:* Medientechnik (Eder et al. 2011), Mediendidaktik (Kerres 2018; Kollar und Fischer 2018; Tulodziecki et al. 2018) und Medienbildung (Tulodziecki 2011)
- *Lehr- und Lernarrangements mit digitalen Medien:* Präsenzangebote mit digitalen Medien, Online-Angebote, Blended-Learning-Angebote
- *Einsatz digitaler Medien: *Medienarten, digitale Werkzeuge, inhaltlich-ausgerichtete und didaktisch-strukturierte Lehr- und Lernangebote (Tulodziecki et al. 2014, 2018)
- *Zugänglichkeit von Lehr- und Lernmaterialien: *kostenpflichtiger und eingeschränkter Zugang, uneingeschränkter offener Zugang wie z. B. bei Open Educational Resources (Blees et al. 2015)

Aus den sechs Einrichtungen liegen für den Erhebungszeitraum September bis Dezember 2019 insgesamt 58 merkmalsgeleitete, halbstandardisierte Interviews mit Expertinnen und Experten (Bogner et al. 2009) vor. Unter Rückgriff auf das dialogische Forschungsformat (Gómez et al. 2011; Tulodziecki et al. 2014) orientiert sich die Operationalisierung des Begriffs „Digitalisierung von Angeboten in der Erwachsenenbildung/Weiterbildung“ an den oben ausgeführten gemeinsam identifizierten Merkmalen. Die Gelingensbedingungen hingegen werden als Eigeneinschätzung der Interviewpersonen operationalisiert: In welcher Weise und unter welchen Bedingungen ist der Einsatz digitale Medien in Angeboten der Erwachsenenbildung/Weiterbildung möglich und werden von den Interviewpersonen als sinnvoll und gewinnbringend eingeschätzt? Mit diesem Zugang erfolgt die Orientierung an einem Verständnis von Gelingen, das sich auch in Evaluationsstudien wiederfindet (z. B. Ehmann 2021; Schulte und Wegner 2021). Daraus ableitend werden mit Perspektive auf die Gelingensbedingungen folgende Fragen aufgeworfen: Werden digitale Medien in Angeboten der Erwachsenenbildung/Weiterbildung überhaupt eingesetzt? Und wenn ja, in welcher Weise? Weiterhin wird danach gefragt, mit welchen (erwachsenenpädagogischen) Erwartungen der Expertinnen und Experten aus der Praxis der Einsatz von digitalen Medien verbunden ist.

Die Audioaufnahmen der Gruppendiskussionen und der Interviews mit Expertinnen und Experten sind transkribiert (Dresing und Pehl 2018) und anonymisiert (Meyermann und Porzelt 2014). Die Analyse der Daten orientiert sich an der Identifizierung von einrichtungsübergreifenden Gelingensbedingungen. Deshalb erfolgte eine fallübergreifende Analyse der Daten, die sich an Prinzipien der qualitativen Inhaltsanalyse nach Mayring (2015) und Kuckartz (2010) orientiert und MAXQDA zur Aufbereitung der Daten nutzt. Die Analyse der aufbereiteten Daten erfolgt im ersten Schritt als deduktive Kodierung der formalen Kategorien entlang der Untersuchungsmerkmale. In einem zweiten Schritt werden auf Ebene der formalen Kategorien anhand einer induktiven Kodierung Gelingensbedingungen als materiale Kategorien identifiziert (Von Hippel 2019). In einem dritten Schritt erfolgt die Entwicklung von axialen Codes. Dieser letzte Schritt zielt auf die Identifikation von Wechselwirkungen (Interdependenzen) zwischen den Ebenen.

## Gelingensbedingungen der Digitalisierung in Angeboten

In den Interviewdaten zeigt sich, dass bei allen Falleinrichtungen das Gelingen der Digitalisierung der Angebote zunächst davon abhängt, ob Personen für die Teilnahme gewonnen werden können. Zum Zeitpunkt der Interviewerhebung war die Coronasituation noch nicht virulent und die Perspektive der Digitalisierung bezog sich auf die Breite der Möglichkeiten von Angeboten mit digitalen Medien. Diese beziehen die Interviewpersonen nicht nur auf Videokonferenzformate, sondern auch auf die Integration digitaler Medien in Präsenzformaten.

Zunächst ist also die Gewinnung von Teilnehmenden in den Blick zu nehmen: Für welche *Adressatinnen und Adressaten* stellen welche digitalen Medien ein attraktives Angebot dar? Die eigenen erwachsenenpädagogischen Anforderungen zeigen sich fast durchgehend in Begründungsformen für den Einsatz digitaler Medien.

### Angebotsentwicklung für Adressatinnen und Adressaten

Mit Blick auf die Angebotsentwicklung stellt die erfolgreiche *Identifikation von digital erreichbaren Adressatinnen und Adressaten* eine wichtige Gelingensbedingung dar. Von den Einrichtungen werden Menschen in sozialen Berufen, Menschen in kaufmännischen Berufen, Menschen mit Fluchterfahrung, Menschen mit körperlichen Einschränkungen und Menschen im Ehrenamt als Gruppen identifiziert, die im Jahr 2019 erreicht werden konnten. Insgesamt wird jedoch an vielen Stellen darauf hingewiesen, dass die Teilnehmenden sich in sehr unterschiedlichen Lebenslagen befinden und die Altersspanne sehr groß ist. Die Benennung der erreichbaren Gruppen kann jeweils nur sehr konkret für ein bestimmtes Angebot und eine bestimmte Region erfolgen.

Für die identifizierten *Adressatinnen und Adressaten* kann sodann die Bereitstellung *bedarfsgerechter Angebote mit digitalen Medien* als weitere Gelingensbedingung identifiziert werden. Eine Interviewperson benennt dies wie folgt: *„ohne Teilnehmende brauchen wir kein Programm zu machen, …“ (C_GD:36) *Das Gelingen bedarfsgerechter Angebote zeigt sich durch die Orientierung an der Nachfrage der Teilnehmenden, aber auch an Anpassung von Zeitformaten und dem sensiblen und an die jeweiligen Teilnehmenden angepassten Einsatz digitaler Medien. Dieser sensible Umgang bezieht sich auf Art und Umfang der eingesetzten digitalen Medien. Eine Einrichtung verweist darauf, dass digitale Medien (z. B. Social Media) auch dazu genutzt werden, um gemeinsam mit potenziellen Teilnehmenden Angebote zu entwickeln und zu planen.

Die Entwicklung von Angeboten mit digitalen Medien verweist darauf, dass sich das Gelingen derselben nicht in der Identifikation von *Adressatinnen und Adressaten* und dem bedarfsgerechten Angebot erschöpft. Vielmehr können die Interviewdaten so interpretiert werden, dass das Gelingen auch von der Erreichbarkeit der *Adressatinnen und Adressaten* abhängt und damit vom Gelingen von Öffentlichkeitsarbeit und Marketing. Es zeigt sich, dass Einrichtungen der Erwachsenenbildung/Weiterbildung stärker als Anbieter von digitalen Formaten wahrgenommen werden müssen. Dies kann als Hinweis auf die Mehrebenenperspektive gedeutet werden. Das Gelingen von Digitalisierung kann nicht einzig über die Ebene der Angebote erfolgen, sondern es bedarf auch der Abstimmung und des Gelingens auf der Ebene der Organisation, indem beispielsweise deren Angebote mit digitalen Medien stärker wahrgenommen werden.

### Medienbezogene Inhalte

In den Interviews zeigt sich weiterhin, dass sowohl Medientechnik, Mediendidaktik als auch Medienbildung Inhalte der Angebote darstellen. *Medientechnik *wird in den klassischen Anwendungsschulungen für Software aufgegriffen. Die Perspektive der *Mediendidaktik* findet sich zum Zeitpunkt der Interview-Erhebung beispielsweise in der Entwicklung von Lernspielen.

*[W]ie können @digitale Spiele@ eingesetzt werden in der Erwachsenenbildung im Kontext politische Bildung, interkulturelle Bildung und religiöse Bildung? Damit befassen wir uns jetzt. (D_GD: 8*)

Das Interview-Zitat deutet auf eine enge Verknüpfung von Digitalisierung mit Entwicklungs- und Pilotierungsperspektiven von Angeboten hin. Die Einrichtungen greifen jedoch auch gezielt Themen von Medienbildung auf, wie beispielsweise ein kritischer Umgang mit Hate Speech, Maschinenlernen und Social Scoring. Die Möglichkeit auf Plattformen oder in Foren über Themen diskutieren zu können, wird zur Stärkung partizipativer Bildung genutzt.

### Lehr- und Lernarrangements mit digitalen Medien

In den untersuchten Einrichtungen der Erwachsenenbildung/Weiterbildung werden fallübergreifend sowohl Präsenzangebote mit digitalen Medien und reine Online-Angebote, als auch Blended-Learning-Angebote als gelingend eingeschätzt. Das Gelingen der Lehr-Lernarrangements mit digitalen Medien wird jeweils didaktisch begründet.

In *Präsenzangeboten mit digitalen Medien* werden diese sehr unterschiedlich eingesetzt und als gelingend erfahren. Die Unterschiedlichkeit bezieht sich auf die Vielfalt digitaler Medien. In den Interviews zeigt sich jedoch auch deutlich, dass die jeweilige Nutzung eines didaktisch geplanten Einsatzes bedarf, damit dieser gelingen kann.

[D]ass man versucht (…) in die Präsenzlehrgänge digitale Inhalte zu integrieren, um einfach auch ein anderes Lernen zu schaffen, dass man die Teilnehmer mehr aktiviert, dass man da auch moderner ist, klar, mit diesen Videos. (H_GD: 36)

Der Einsatz digitaler Medien in Präsenz-Angeboten wird von den Interviewpartnern durchgängig dann als gelingend eingeschätzt, wenn dieser didaktisch begründet erfolgt. Dazu zählt insbesondere die Aktivierung von Teilnehmenden.

Zum Zeitpunkt der Interview-Erhebung im Jahr 2019 wurden *reine Online-Angebote* in den Falleinrichtungen in eher geringerem Umfang angeboten. An wenigen Stellen wurde jedoch von einem gelungenen reinen Online-Angebot berichtet. Die Interviewdaten deuten darauf hin, dass digitale Medien als didaktische Elemente genutzt werden, um Seminarthemen in verschiedenen Formen präsentieren zu können.

Diese Kurse [zu betriebswirtschaftlichen Themen, Anm. d. V.], die finden ausschließlich als Webinar statt, einmal in der Woche abends. Und wenn man dann online sich nicht dazu schalten kann, zum Live-Webinar, dann kann man es/ein paar Tage später kann man sich […] das Ganze angucken. Das Ganze wird flankiert noch mit Lehrmaterial in Buchform sozusagen, da kann man alles nachlesen. (B_I2: 154)

Perspektiven von synchroner und asynchroner Verfügbarkeit der Seminarmaterialien verweisen auf die hohen Flexibilitätsoptionen im Lernprozess. Die zeitliche Verfügbarkeit der Teilnehmenden sollte demnach berücksichtigt werden.

Die größte Variation an Lehr- und Lernarrangements mit Medienunterstützung findet sich in den Interviewdaten bei den *Blended-Learning-Angeboten*. Über alle Formen der Blended-Learning-Angebote hinweg finden sich didaktische Begründungen, weshalb einige Teile in Präsenzform und andere online angeboten werden. Dazu entwickeln die Einrichtungen strukturierte Zeitpläne und Lernwege für Präsenzphasen, Online-Phasen, Studienmaterialien und Aufgaben. Präsenzphasen werden beispielsweise für Erläuterungen und die Planung von Online-Phasen oder für persönliche Begegnungen und das Kennenlernen genutzt.

[W]ir haben Präsenz, man lernt sich kennen, man kann hinterher auch durch das gewonnene Vertrauen miteinander nochmal im Chat Fragen weiter miteinander auch bearbeiten. (D_GD: 56)

Präsenzphasen unterstützen damit die sozialen Aspekte des Lernens auch während der Online-Phasen. Online-Phasen werden genutzt, um Inhalte zu erarbeiten, zu vertiefen, einzuüben oder zur Unterstützung selbstgesteuerter Lernstandskontrollen. Für die Durchführung der Online-Phasen arbeiten die Falleinrichtungen auch mit individueller Lernbegleitung. Die Lernbegleitung dient der Unterstützung bei der zeitlichen Strukturierung und bei inhaltlichen und technischen Fragen. Die Interviews deuten darauf hin, dass die individuelle Lernbegleitung vor allem dann notwendig ist, wenn es nur wenige Präsenzphasen gibt. Spannend zu beobachten ist jedoch auch, dass Online-Phasen als optionale Ergänzung zu Präsenz-Angeboten entwickelt werden. Dies erfolgt beispielsweise mit der Bereitstellung von selbstgesteuerten Lernstandskontrollen, die Teilnehmende beim Besuch einer Aufstiegsfortbildung und zur Vorbereitung auf eine Prüfung nutzen können. Eine andere Form von Blended-Learning-Angeboten stellen hybride Angebote dar. Diese wurden im Jahr 2019 bereits von Einrichtungen genutzt, die standortübergreifend Weiterbildungsangebote bereitstellen, und damit Teilnehmende in Einrichtungen an unterschiedlichen Standorten über Videokonferenzsysteme zusammenbringen.

### Einsatz digitaler Medien

Mit Blick auf die *digitalen Medienarten* findet sich in den Interviews der Verweis auf Laptops mit Beamer, Dokumentenkameras, interaktive Boards und an einer Stelle auch auf VR-Brillen. Der Einsatz digitaler Medienarten scheint stark an die Verfügbarkeit in den Seminarräumen und Einrichtungen gebunden zu sein. Mit Blick auf die Interviews zeigt sich, dass die Einrichtungen insbesondere dann, wenn sie über keine eigenen Räumlichkeiten verfügen, beim Einsatz von digitalen Medienarten stark von der Ausstattung des Raumes abhängig sind. Hier stellt sich die Herausforderung, wie die Ausstattung von beispielsweise kommunal genutzten Seminarräumen mit Einrichtungen der Erwachsenenbildung/Weiterbildung abgestimmt werden kann.

In den Interviews findet sich eine Vielzahl an *digitalen Werkzeugen*, deren Einsatz als gelingend eingeschätzt wird. Bei der Software wird häufig auf Standardsoftware (z. B. Präsentationssoftware oder E‑Mailprogrammen) zurückgegriffen. Der gelungene Einsatz wird jeweils mit einem bestimmten didaktischen Zweck verbunden. Darüber hinaus werden im Bereich der digitalen Werkzeuge auch Learning- und Content-Management-Systeme oder Videokonferenzsysteme, die für bestimmte didaktische Ziele genutzt werden können, eingesetzt. Sehr umfangreich werden Online-Tools eingesetzt und auch als gelingend eingeschätzt: Online-Tools lassen sich differenzieren in solche zur Erstellung von Quiz (z. B. Kahoot), zur Erstellung virtueller Pinnwände (z. B. Mural oder Miro), für virtuelle Umfragen (z. B. Mentimeter), zum Wissensmanagement (z. B. Trello) oder zur Erstellung von Lernbausteinen (z. B. Learning Snacks). Auch hier zeigen sich im Interviewverlauf didaktische Begründungen, die insbesondere auf die Partizipation von Teilnehmenden im Seminarverlauf zielen.

Mit Blick auf *didaktisch-strukturierte digitale Medienangebote* findet sich in den Interviews sowohl der Einsatz von bereits verfügbaren als auch die Neuentwicklung von didaktisch-strukturierten digitalen Medienangeboten. Beispiele für den Einsatz von bereits verfügbaren didaktisch-strukturierten digitalen Medienangeboten stellen digitale Lehrprogramme oder digitale Lehrbücher dar, die insbesondere in Sprachangeboten durch Verlage zur Verfügung gestellt werden. Aber auch Experimentier- und Simulationsumgebungen werden im Bereich Kultur genutzt. Hier wird auf digitale Ressourcen aus Museen oder Galerien zurückgegriffen. Der Einsatz von (Erklär‑)Videos, die über Videoplattformen zur Verfügung stehen, verweist auf die Nutzung von bereits bestehenden didaktisch-strukturierten digitalen Medienangeboten. In den Interviews zeigt sich jedoch auch, dass viele Einrichtungen selbst didaktisch-strukturierte digitale Medienangebote entwickeln, um passgenaue Angebote für ihre Zielgruppen zu schaffen. Dazu zählt beispielsweise die Entwicklung von Lehrprogrammen. Hier werden aus traditionellen Studienbriefen Lehrprogramme entwickelt, die die Breite des Einsatzes digitaler Medien, wie z. B. den Einbezug von digitalen Texten, Audioausschnitten, Videos und Lernspielen mit planen. Didaktisch-strukturierte digitale Medienangebote werden auch im Bereich der Aufstiegsfortbildung entwickelt, um beispielsweise Übungsprogramme zielgenau für die Prüfungsvorbereitung einzusetzen. Aber auch digitale Lernspiele werden für den passgenauen Einsatz in der Erwachsenenbildung/Weiterbildung entwickelt. Insgesamt zeigt sich in den Interviews ein breites Repertoire an genutzten didaktisch-strukturierten digitalen Medienangeboten. Weniger zu finden sind Augmented- oder Virtual-Reality-Anwendungen und intelligente tutorielle Systeme. Dies kann mit den noch gering verfügbaren Angeboten und der kostenintensiven Entwicklung begründet werden. In den Interviews deutet sich übergreifend an, dass der Einsatz digitaler Medien dann als gelingend eingeschätzt wird, wenn er didaktisch an die jeweilige Seminarsituation angepasst und den Bedarfen der jeweiligen Zielgruppen genau gerecht werden kann. Gleichzeitig zeigt sich jedoch auch die Herausforderung der Entwicklung und des Zugangs zu digitalen Medien, da an vielen Stellen Entwicklungen mit Kosten verbunden sind. Um diese Kosten zu stemmen, zeigen sich Kooperationen und Netzwerke zwischen Einrichtungen und Mitarbeitenden als Bedingung, die zum Gelingen von Digitalisierung beitragen können. Die Kooperationen und Netzwerke sind nicht nur innerhalb der Erwachsenenbildung/Weiterbildung notwendig, sondern auch im Hinblick auf andere Bildungseinrichtungen (z. B. Schulen zur gemeinsamen Nutzung von Medienarten) und Anbieter von digitalen Lehr- und Lernmaterialien (z. B. Verlage).

### Zugänglichkeit von digitalen Lehr- und Lernmaterialien

Der Einsatz didaktisch-strukturierter digitaler Medienangebote ist verbunden mit dem Zugang zu den relevanten digitalen Lehr- und Lernmaterialien.

In den Interviewdaten zeigt sich, dass zunächst *bereits verfügbare Lehr- und Lernmaterialien *genutzt werden. Diese stellen beispielsweise Materialien und Medienangebote dar, die von Verlagen insbesondere für Sprachangebote zur Verfügung gestellt werden. Darüber werden Lehr- und Lernmaterialien eingesetzt, die Museen für Angebote im Bereich Kultur zur Verfügung stellen, Erklärvideos, die auf frei zugänglichen Plattformen bereitstehen und frei zugängliche Online-Wörterbücher im Bereich Sprachen.

Eine weitere Form der Zugänglichkeit zu digitalen Lehr- und Lernmaterialien stellt der *Aufbau von gemeinsamen Pools mit digitalen Lehr- und Lernmaterialien* dar. Der gemeinsame einrichtungsübergreifende Pool von digitalen Lehr- und Lernmaterialien ermöglicht Mitarbeitenden und Mitwirkenden der beteiligten Einrichtungen, selbst Materialien zu teilen und andere Materialien zu verwenden.

Eine weitere Form der Zugänglichkeit stellen *in Seminarkontexten erstellte Videos* dar. Die Videos entstehen in sehr unterschiedlicher Weise. So werden beispielsweise Videoaufnahmen mit Beiträgen von Expertinnen und Experten erstellt. Ein Interviewzitat verweist auf die gemeinsame Erstellung von Erklärvideos mit Teilnehmenden, ein anderes Interviewzitat auf die Erstellung von Erklärvideos durch Mitarbeitende.

## Fazit

Mit Blick auf die Frage, welche Angebote mit digitalen Medien in den Einrichtungen der Erwachsenenbildung/Weiterbildung gelingen, zeigt sich in den Daten durchgängig, dass ein umfangreicher variantenreicher Einsatz von digitalen Medienarten, digitalen Werkzeugen und digitalen Medienangeboten stattfindet und von den Interviewpersonen als gelingend eingeschätzt wird. Es zeigen sich keine klaren Einsatztrends, welche Lehr-Lernarrangements mit digitalen Medien durchgängig bei allen Teilnehmenden gelingen. In den Begründungen der Interviews finden sich vielmehr genauere Hinweise darauf, dass das Gelingen von einer passgenauen Abwägung des Einsatzes für die jeweiligen Adressatinnen und Adressaten sowie Teilnehmenden abhängig ist. Überall dort, wo Angebote mit digitalen Medien in der Erwachsenenbildung als gelingend eingeschätzt werden, gibt es genaue Überlegungen, welche digitalen Medien in welcher Weise, für welche Adressatinnen und Adressaten und mit welchem Ziel eingesetzt werden. Der Einsatz digitaler Medien in Angeboten der Erwachsenenbildung/Weiterbildung stellt somit eine Erweiterung der Orientierung an Adressatinnen und Adressaten um die Perspektive der Digitalisierung dar. Die erwachsenenpädagogisch-didaktische Arbeit erweitert sich durch den Einsatz digitaler Medien grundlegend und wird anspruchsvoller. Bestehende didaktische Konzepte können nicht repetitiv genutzt werden. Vielmehr bedarf es einer grundlegenden didaktischen Neu- oder Weiterentwicklung von Angeboten, die um den Einsatz digitaler Medien ergänzt ist.

Damit diese Abwägungen erfolgen können, sind die Verfügbarkeit und der einfache Zugang zu digitalen Medienarten, digitalen Werkzeugen und didaktisch-strukturierten digitalen Medienangeboten notwendig. Dazu zählen zum Beispiel interaktive Tafeln, Lernmanagement-Systeme, Videokonferenzsysteme oder digitale Lehr-Lernmaterialien. Bei der Auswahl von digitalen Medienarten, digitalen Werkzeugen und didaktisch-strukturierten Medienangeboten zeigt sich, dass die einfache technische Handhabung zentral für den gelingenden Einsatz ist. Die Interviews deuten darauf hin, dass bereits kleine technische Herausforderungen bei Mitarbeitenden, Mitwirkenden, Dozierenden und/oder Teilnehmenden zu großen Einschränkungen des Gelingens führen können. Dazu zählt auch die Verfügbarkeit einer schnellen Internetverbindung.

Die Interviewdaten deuten darauf hin, dass der Einsatz digitaler Medien in Angeboten ein Anlass ist, sich weiter mit der Frage von Digitalisierung in der Erwachsenenbildung/Weiterbildung zu beschäftigen. Diese Beschäftigung bezieht sich beispielsweise auf kritisch-reflexive Überlegungen im Bereich der Medienbildung, aber auch auf das Potenzial für die teilnehmendengerechte Weiterentwicklung des Einsatzes von digitalen Medien in Lehr- und Lernangeboten.

Der Beitrag untersucht die Digitalisierung in Angeboten durch klassische Einrichtungen der Erwachsenenbildung/Weiterbildung. Die betriebliche Weiterbildung wird anhand des vorliegenden Samples nicht berücksichtigt. In der betrieblichen Weiterbildung ist eine große Abhängigkeit von der Branche und Größe der Unternehmen zu erwarten. Zudem ist hier eine größere Homogenität der Adressatinnen und Adressaten zu erwarten. Das Sample nimmt auch keine Anbieter in den Blick, die Erwachsenenbildung/Weiterbildung ausschließlich in digitalisierter Form anbieten oder die aus dem Bereich der Digitalunternehmen (z. B. Google, LinkedIn) kommen. Vielmehr gehören die untersuchten Einrichtungen zu Dachverbänden der großen Anbieter von Erwachsenenbildung/Weiterbildung in Deutschland, die ihre Angebote seit Jahrzehnten mit und ohne digitale Medien anbieten. Deshalb impliziert die Untersuchung den Wandel der Angebote in der Erwachsenenbildung/Weiterbildung durch die Möglichkeiten der Digitalisierung. Gleichzeitig ist zu betonen, dass die Analyseergebnisse vor allem die Einrichtungen in den Blick nehmen, die vor der Pandemie über eine hohe Affinität zur Nutzung von digitalen Medien verfügen. Es ist bereits absehbar, dass sich durch die Pandemie große Veränderungen ergeben. So unterliegen Digitalisierungsentwicklungen durch die Kontaktbeschränkungen der Pandemie anderen Erfordernissen und Bedingungen als vor der Pandemie.

Insgesamt zeigt sich, dass das Gelingen von Digitalisierung in Angeboten der Erwachsenenbildung/Weiterbildung stark vom Gelingen didaktischer Entwicklungen abhängt. Digitalisierung erhöht die didaktischen und erwachsenenpädagogischen Anforderungen in der Angebotsentwicklung. Als Konsequenz erhöht der Einsatz digitaler Medien die didaktische Entwicklungsarbeit, die sich sowohl auf die vorausschauende Planung des Einsatzes als auch die situative Anpassung im Angebotsgeschehen bezieht.

## Footnotes

1 Egetenmeyer, R. und Grafe, S. (2017). *Digitalisierung in der Erwachsenenbildung und beruflichen Weiterbildung.* Unveröffentlichter Antrag für eine BMBF-Projektförderung im Programm Digitalisierung im Bildungsbereich. Würzburg: Universität Würzburg.

2 Egetenmeyer, R. & Grafe, S. (2017). *Digitalisierung in der Erwachsenenbildung und beruflichen Weiterbildung.* Unveröffentlichter Antrag für eine BMBF-Projektförderung im Meta-Vorhaben Digitalisierung im Bildungsbereich. Würzburg: Universität Würzburg.

3 Die Literaturanalyse folgte einem breiten Rechercheansatz, der sowohl Fachzeitschriften der Erwachsenenbildung (z. B. Hessische Blätter für Volksbildung, Zeitschrift für Weiterbildungsforschung), als auch Datenbanken (z. B. FIS-Bildung, Forschungslandkarte des Dt. Instituts für Erwachsenenbildung) einbezog. Auch die Schlagwortsuche war breit angelegt mit Schlagworten aus der Medienpädagogik (eLearning, Medienkompetenz, digitales Lernen, neue Medien, digitale Medien, digitale Bildung), die mit den Begriffen Erwachsenenbildung, Weiterbildung und Lebenslanges Lernen kombiniert wurden bzw. hinsichtlich deren Relevanz gefiltert wurde. Für die Literaturanalyse ist eine Citavi-Datenbank angelegt worden, in der die Literatur merkmalsgeleitet zugeordnet ist.
